# Digital phenotyping for classification of anxiety severity during COVID-19

**DOI:** 10.3389/fdgth.2022.877762

**Published:** 2022-10-13

**Authors:** Binh Nguyen, Martin Ivanov, Venkat Bhat, Sri Krishnan

**Affiliations:** ^1^ Signal Analysis Research (SAR) Group, Department of Electrical, Computer, and Biomedical Engineering, Toronto Metropolitan University, Toronto, ON, Canada; ^2^ Interventional Psychiatry Program, St. Michael’s Hospital, Department of Psychiatry, University of Toronto, Toronto, ON, Canada

**Keywords:** digital phenotyping, machine learning, COVID-19, anxiety, mental health

## Abstract

COVID-19 has led to an increase in anxiety among Canadians. Canadian Perspectives Survey Series (CPSS) is a dataset created by Statistics Canada to monitor the effects of COVID-19 among Canadians. Survey data were collected to evaluate health and health-related behaviours. This work evaluates CPSS2 and CPSS4, which were collected in May and July of 2020, respectively. The survey data consist of up to 102 questions. This work proposes the use of the survey data characteristics to identify the level of anxiety within the Canadian population during the first- and second-phases of COVID-19 and is validated by using the General Anxiety Disorder (GAD)-7 questionnaire. Minimum redundancy maximum relevance (mRMR) is applied to select the top features to represent user anxiety, and support vector machine (SVM) is used to classify the separation of anxiety severity. We employ SVM for binary classification with 10-fold cross validation to separate the labels of *Minimal* and *Severe* anxiety to achieve an overall accuracy of 94.77±0.13% and 97.35±0.11% for CPSS2 and CPSS4, respectively. After analysis, we compared the results of the first and second phases of COVID-19 and determined a subset of the features that could be represented as pseudo passive (PP) data. The accurate classification provides a proxy on the potential onsets of anxiety to provide tailored interventions. Future works can augment the proposed PP data for carrying out a more detailed digital phenotyping.

## 1. Introduction

Mental health is one of the greatest inequalities in terms of prevalence across the globe, with up to 80% of cases involving some sort of psychosis conditions occurring in low- and middle-income countries (1). Treatment for mental health disorders are consistently expensive among countries around the world (2). This can cause inequality and unequal access to mental health treatments for patients in poorer countries. Studies on mental health disorders in low- and middle-income countries have been recognized (3, 4), allowing for a better understanding of mental health applications in subpopulations. The opportunity to apply digital phenotyping applications can offer low-cost aid for diagnosis of mental health disorders and digital interventions (5, 6).

There are various aspects that can affect a person’s mental health, including internal and external factors. Internal factors include physical health and genetic predisposition (7), whereas external factors include financial insecurity, food insecurity, and lifestyle changes (8). Mental health is an obscure topic as it can affect everyone personally (9). Due to the COVID-19 pandemic, there has been a deterioration in the general public’s mental wellbeing, causing an increase in discussions related to mental health (10, 11).

The main aim of this work is to identify characteristics from the Canadian Perspective Survey Series (CPSS) (12) data to evaluate the level of anxiety within the Canadian labour force population. The CPSS dataset is a series of datasets collected by Statistics Canada and is used to evaluate the physical and mental health of Canadians at different stages of the COVID-19 pandemic. This work focuses on the Canadian Perspectives Survey Series 2, 2020: Monitoring the Effects of COVID-19 (CPSS2) and the Canadian Perspective Survey Series 4, 2020: Information Sources Consulted During the Pandemic (CPSS4), to evaluate the mental health of users within the Canadian labour force population. These datasets were collected online in May and July, respectively. CPSS2 was collected during May 2020, and the purpose of this dataset was to survey the mental and physical health effects of the COVID-19 pandemic on Canadians. CPSS2 was associated with the beginning of the first lockdown (12, 13). CPSS4 was the subsequent dataset of the series, which was collected during July in 2020 (14). CPSS4 is a continuation of CPSS2, in addition to collecting information about the sources consulted during the pandemic. This dataset was associated with the end of the first lockdown (13, 14). The labour force is broken down into two sections, namely, the employed and unemployed population. The employed are defined as persons holding a job or owning a business, and the unemployed are defined as those without work and actively seeking work.

The current literature uses the CPSS dataset to evaluate user anxiety through self-perceived mental health. We hypothesize a methodology that can indirectly assess self-perceived anxiety through the successful identification of survey data characteristics. Instead of the general self-perceived mental health response labels used in Findlay et al. (10) and Zajacova et al. (15), we propose the use of the more quantified General Anxiety Disorder (GAD)-7 labels to assess anxiety among the general public during the COVID-19 pandemic. Using the GAD-7 severity levels, we harness the novel feature selection and machine learning classification techniques to better understand what contributes to anxiety and how to provide early interventions.

This work aims to study the use of survey data to influence the future of Ecological Momentary Assessment (EMA) in mental health. EMA is the sampling of a subjects’ current behavior and experiences in real time (16). It is typically sampled in their natural environment. This work uses CPSS, where the survey questions are sampled throughout the pandemic. This work is used to analyze the characteristics of the CPSS dataset to successfully evaluate the anxiety of the Canadian population. Once successfully evaluated using the CPSS data, the results of this paper can be used in future work to offer improved and efficient data collection. This will allow continuous monitoring and monitor the trends of user anxiety (17).

The rest of this paper is organized as follows: Section 2 presents a literature review of the key related works. Section 3.1 discusses the CPSS data in further detail and Section 3.1.4 presents the methodologies used for feature selection and classification. Finally, the results are presented in Section 4 with a discussion on the conclusionsdrawn in Section 5.

## 2. Related works

Studies that have involved mental health research during COVID-19 include the work by Dagklis et al. (18). This work focuses on the perinatal of mental health during lockdown in Greece. The motivation for this work stems from the hypotheses of previous pandemics (SARS and MERS) that pregnant women were more likely to be psychologically affected (19, 20), which could lead to potential negative consequences on perinatal outcomes (21). To quantitatively monitor perinatal anxiety and depression, the State–Trait Anxiety Inventory and the Edinburg Postnatal Depression Scale are used (22, 23). This study followed the State–Trait Anxiety Inventory and Edinburg Postnatal Depression Scale score ranges and cut-offs. A total of 269 women consented to participate in the study. The results revealed that 37.5% of the participants experienced a state anxiety score of 42 (mild anxiety) and 13.0% of particpants experienced a trait anxiety score of 35 (no anxiety) (18). The State–Trait Anxiety Inventory scores were assessed during weeks 1, 3, and 6, and it was discovered that participants had feelings of tension, strain, and confusion. During week 6, they were feeling more frightened. The mass quarantine negatively affected the anxiety levels of the majority of pregnant women in Greece. Given these examples, it is evident that the COVID-19 lockdown has had a negative effect on mental health, regardless of geographical location.

In addition to these effects, the COVID-19 pandemic is having a significant socioeconomic impact on the vast majority of the general public (10). CPSS is a series of surveys undertaken by Statistics Canada, which assesses the impacts of the COVID-19 pandemic on the Canadian labour force (12). A few studies have been conducted on CPSS using perceived mental health categories (10, 15). These perceived mental health labels are *Excellent, Very Good, Good, Fair, and Poor*. CPSS contains questions asking about individual impressions of the pandemic from both the health and the economic standpoint. The questionnaire clearly pertains to mental health, as evident from the fact that it asks numerous questions in regards to the self-perceived mental health and causalities associated with positive and negative self-assessments. In particular, the GAD-7 questionnaire is one such metric validated by the Diagnostic and Statistical Manual of Mental Disorders (DSM) for the rating of anxiety severity (24–26). The representation of GAD-7 is a more quantified measure of the severity of anxiety as illustrated in Figure 3. Perceived mental health has traditionally been used as the standardized label for mental health studies. Polsky and Gilmor utilized self-perceived mental health to compare food insecurity among Canadians during the COVID-19 pandemic (8). This study used logistic regression with sociodemographic covariate adjustment. Based on the study, individuals with moderate food insecurity experienced three times higher odds of reporting lower levels of mental health and higher levels of anxiety. When compared with individuals with severe food insecurity, the ratios for mental health and anxiety increased to 4 and 7.6, respectively.

In a similar work, Bulloch et al. (27) used CPSS2 to determine that the COVID-19 pandemic was associated with a decrease in mental health in those under the age of 65. The evaluation was estimated through the use of self-reported mental health and GAD questionnaires. In an article by Lin (28), it is revealed that the author used CPSS4 and extracted information about GAD-7, exposure to COVID-19 misinformation, records of precarious employment, and health behaviour changes to explore gender-specific mental health during the pandemic. It was determined that anxiety levels differed between male and female participants. It was discovered that female participants experienced twice the prevalence of moderate-severe scores of anxiety on the GAD-7 survey (17.2% to 9.9% for female to male, respectively, p<0.001) (28).

In other studies that have used CPSS datasets for analysis, it is revealed that Nguyen et al. (29) utilized GAD-7 scores, from the CPSS2 dataset, as a label identifying indicators of anxiety in Canadians at the beginning of the first lockdown in Canada. CPSS2 comprises 62 questions, and the author employed minimum redundancy maximum relevance (mRMR) to reduce the feature set to the top 20 features. Hierarchial classification was implemented and a support vector machine (SVM) binary classification with 10-fold cross validation was employed to classify *Minimal* and *Severe* anxiety to achieve an overall accuracy of 94.77%. This work proposes the term pseudo passive (PP) data, which can be considered active data that can be augmented as passive data. There are many potential benefits in PP data such as reduction in survey fatigue and passive data collection (29).

The adoption of the collecting PP data through the use of digital platforms and wearables allows for different perspectives for affective computing and digital phenotyping. Affective computing is defined by the study of emotional states through the use of technologies such as systems and devices, which recognize, interpret, process, and simulate emotion (30). This is a multidisciplinary field that encompasses engineering, computer science, psychology, sociology, cognitive science, and others. Moreover, digital phenotyping is defined by Torous et al. (31) as the moment-by-moment evaluation of personalized human phenotype through the use of smartphone and digital devices. The data collected have two subgroups consisting of passive and active data. There have been only a limited number of studies that have used machine learning or statistical analysis to classify mental health from active, passive, and PP data. Studies that have incorporated the techniques and data streams to identify mental health markers include (32–35).

StudentLife project is a publicly available dataset collected at the Dartmouth College (32) that contains active and passive data from 60 participants over 10 weeks. Studies by Farhan et al. (34) and Nguyen et al. (33) have used the StudentLife dataset to apply techniques such as multiview biclustering and decision tree (DT) classification to classify depression severity and have achieved overall classification accuracies of 87.1% and 94.7%, respectively.

In similar studies, Melcher et al. (35) collected passive and active data from college students to determine how digital biomarkers of behavior correlate with mental health. Statistical analysis was conducted and it discovered a correlation of sleep variance with depression scores (p=0.28) and stress scores (p=0.27).

Currently, EMA data can be collected using smartphones for affect and stress assessments (36). We believe that a subset of this EMA data, which still requires active engagement from users for responses, can be substituted with PP data collection. An example of the aforementioned includes “What type of physical activity are you doing right now?” (37). This EMA can be replaced by PP by using an accelerometer (29).

Studies by Curtis et al. (38) and Rivenbark et al. (39) have examined census data collected from Scotland and the USA, respectively, to evaluate the mental health of the target population. Similarly, this paper aims to analyze correlates of anxiety symptoms among the Canadian labour force in the CPSS dataset. In doing so, the term PP can be further developed, creating a foundation for future studies to potentially use PP in the replacement of EMA and active data collection. This has the potential to advance the field of digital phenotyping, offering users more flexibility to collect data.

## 3. Methods

### 3.1. Dataset

Presently, the CPSS dataset comprises six series, collected in April, May, June, July, and September of 2020 and January of 2021. The datasets used in this paper are CPSS2 (12) and CPSS4 (14). The study has a total of 31,896 user sign-ups, which are divided between the six series, and has a participation rate of 23%.

The target populations of these surveys are Canadians that are 15 years or older and part of the labour force, with the exception of full-time members of the Canadian Armed Forces. One participant per household is randomly selected to engage in CPSS. The purpose of the data collection exercise is to obtain information from the participants about any alterations that they experienced in their health condition and in their health behaviours during the COVID-19 pandemic.

#### 3.1.1. First phase of COVID-19

CPSS2 was collected between May 4, 2020, and May 10, 2020. We will refer to this as the first phase of COVID-19 as it encompasses the start of the first wave and beginning of the lockdown. This dataset had 7,242 eligible participants, of whom 4,600 responded at a rate of 63.5%. This series contained 62 variables that were grouped into Behaviour (BH), Demographics (DEM), Derived Variables (DV), Food security (FSC), Labour market impacts (LM), Mental health impacts (MH), and Survey related variables (SRV). The groups BH, DEM, DV, FSC, LM, MH, and SRV contain 29, 9, 4, 1, 8, 8, and 3 variables, respectively.

**Figure 1:**
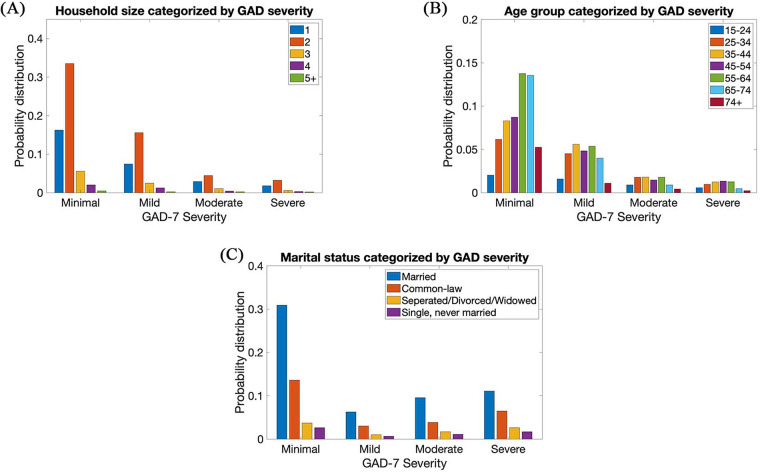
(**A**) Household, (**B**) age group, and (**C**) statistics of subjects in CPSS2.

Figure 1 visualizes the probability distribution of the demographics (household, age group and marital status) of participants in the first phase of the pandemic in respect to the severity of anxiety. In the CPSS2 dataset, it is revealed that 76% and 49.4% of the participants were born in Canada and were male, respectively, while the remaining participants were not born in Canada and are female, respectively.

#### 3.1.2. Second phase of COVID-19

CPSS4 was collected from July 20, 2020, until July 26, 2020, and we will refer to it as the second phase of COVID-19. This dataset had 7,242 eligible participants, with 4,218 responding at a rate of 58.2%. This series contained 102 variables that were grouped into BH, DEM, MH, SRV, Checking Information Sources (FC), and People in Contact (PBH). The groups BH, DEM, MH, SRV, FC, and PBH contained 45, 10, 12, 3, 30, and 2 variables, respectively.

**Figure 2:**
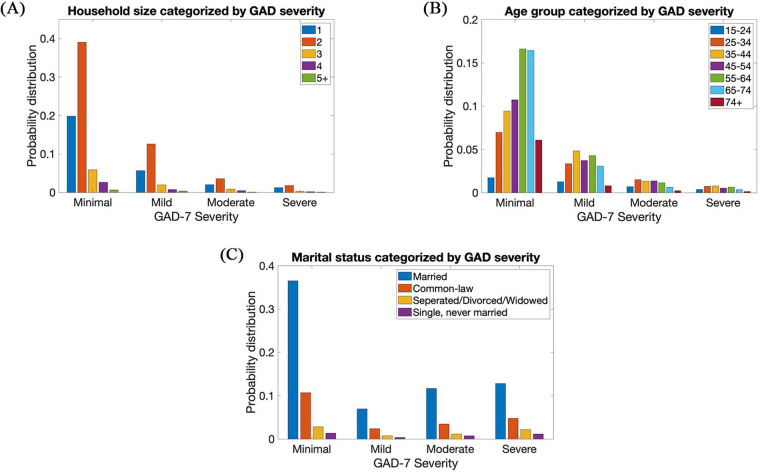
(**A**) Household, (**B**) age group, and (**C**) statistics of subjects in CPSS4.

Figure 2 visualizes the probability distribution of the demographics (household, age group, and marital status) of participants in the second phase of COVID-19 in respect to the severity of anxiety. In the CPSS4 dataset, 84% and 46.1% of the participants were born in Canada and are male, respectively. While the remaining participants were not born in Canada and are female, respectively.

#### 3.1.3. GAD-7

This paper chooses to focus on the first and second phases of COVID-19 as these are the only series that contains mental health survey questions, which include perceived mental health and GAD-7. GAD-7 determines the severity of anxiety disorder based on a self-diagnostic survey. The survey questions are scored between 0 and 3 and consist of seven questions totalling to a max score of 21 (24). The survey has four levels of anxiety severity, namely, *Minimal*, *Mild*, *Moderate*, and *Severe Anxiety*, these are determined by the score cut-off points of 5, 10, and 15, respectively (24).

#### 3.1.4. Demographics

In CPSS2, the demographic information collected included household size, the age of the respondent, immigration status, the sex of the respondent, the presence of the dependent child as of May 4, 2020, the marital status of the respondent, the type of dwelling, the highest level of education completed, and rural/urban indicators. Similarly, CPSS4 collected the same demographic information, in addition to the employment status of the respondent. Due to the anonymization of the data, the survey response relationship for each user could not be tracked. This made it difficult to create a direct relationship of any findings with subpopulations. Instead, the findings could be generalized to the general Canadian population.

It could be seen that households categorized by GAD severity had a right skewed distribution where a small household size dominates each category of severity. However, age groups categorized by GAD had a Gaussian-type distribution where the age groups were distributed evenly across each category of severity. Lastly, it could be seen that trends in the first and second phases of COVID-19 were very similar. Although very minimal, it could be seen that there are less instances of the severe category and more instances of minimal category in the second phase of COVID-19 than in the first phase of COVID-19.

### 3.2. Pre-processing

Prior to analysis, the GAD-7 metric data were pre-processed. Pre-processing involved the removal of GAD-7-related features that were directly related to the survey due to the GAD-7 severity metric being used as the class label (ANXDVSEV column header). The GAD-7-related features that were removed were seven questions consisting of GAD (MH15A, MH15B, MH15C, MH15D, MH15E, MH15F, MH15G), GAD score (ANXDVGAD), and GAD cut-off (ANXDVGAC). Further pre-processing was conducted in order to remove any data samples where a GAD-7 severity metric response was not provided. The data was then normalized using min–max normalization (40). The normalization equation is represented in Equation 1, where x represents the respective feature column

(1) $$x^{\prime }=\frac{x-\max(x)}{\max(x)-\min(x)}$$

### 3.3. Feature learning

The full list of features can be seen in Statistics Canada (12, 14). To identify the significant features of the data, we applied feature learning techniques. Two feature learning tasks were employed and we found that mRMR provided the best outcome.

#### 3.3.1. Minimum redundancy maximal relevance

For feature selection, the mRMR algorithm was proposed. This approach optimizes the mutual information values, represented as I(x;y), where x and y represent the random variable (41). The aim of this approach is to maximize the distance Φ between the max-dependency and min-redundancy as in Equation 2. However, due to the computational cost of maximum dependency, a simpler approximation was introduced, which was maximum relevance. Maximum relevance (D) between the subset of features $x_{i}\in S$ and the target class c was obtained as in Equation 3. Redundancy estimation for features was calculated by using mutual information values between two features. Minimum redundancy R calculation is provided in Equation 4.

(2) maxΦ(D,R),Φ=D−R

(3) $$\max D(S,c),\;D=\frac{1}{\left|S\right|}\sum_{x_{i}\in S}I(x_{i};c))\;$$

(4) $$\min R(S),\;R=\frac{1}{|s{|}^{2}}\sum_{x_{i},x_{j}\in S}I(x_{i};x_{j})\;$$

#### 3.3.2. Relieff feature learning

Another feature learning algorithm that was applied was Relieff (42). This algorithm was proposed by Kira and Rendell (42) to enhance learning times and the accuracy of learned concepts. The original algorithm was proposed for binary classification but is possible for multinomial classification by decomposition into a number of binary problems. Given the feature set F as $\left\{f_{1},f_{2}\ldots f_{k}\right\}$ with instance X denoted by the k-dimensional vector $\left\{x_{1},x_{2}\ldots x_{k}\right\}$, the Relieff algorithm was used to detect features that are statistically relevant to the target concept. The feature vector was iterated m times and the near-hit and near-miss values were calculated by the p-dimensional Euclid distance. The near-hit and near-miss values were used to update the weight vector W with index i, which is represented in Equation 5. The feature weight was calculated for every triplet sample, which is also known as relevance. Lastly, relieff selected relevance values that were above a given threshold τ.

(5) $$W_{i}=W_{i}-(x_{i}-{\mathrm{nearHit}}_{i}{)}^{2}+(x_{i}-{\mathrm{nearMiss}}_{i}{)}^{2}\;$$

### 3.4. Label separation

We separated the label to evaluate four cases. The case separations were proposed to enhance the understanding of classifying GAD within the Canadian labour force population during the first and second phases of COVID-19. Preliminary verification of the selected features was achieved using the greatest distance between the labels, i.e., *Minimal* and *Severe Anxiety*.

**Figure 3:**
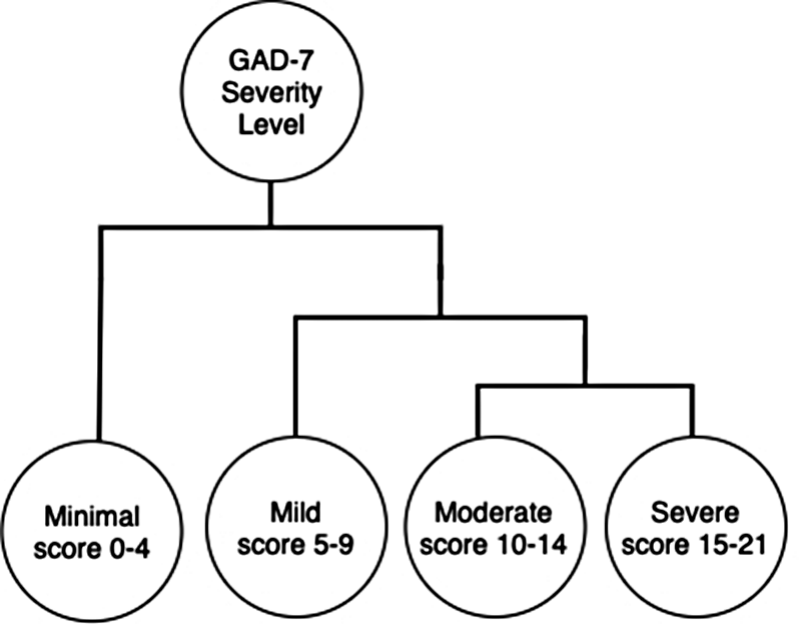
Hierarchical depiction of the GAD-7 severity levels with cut-off scores.

The second case involved a more granular separation between adjacent labels, where hierarchical grouping were used to further test the robustness of these representative features. We followed GAD-7’s hierarchical structure illustrated in Figure 3 for the robustness studies.

The third case was a binary classification with a GAD score of 10. The significance of the score of 10 was suggested to be a reasonable cut-off for identifying cases of GAD (24). In a study by Spitzer et al. (24), 965 patients conducted a telephone interview with a mental health professional to determine the presence of GAD diagnosis. It was determined that the cut-off of ≥10 was significant, as it is an optimal balance between sensitivity (89%) and specificity (82%) of GAD symptoms (24, 43).

Lastly, the labels will be separated into its respective classes of *minimal, mild, moderate, and severe*. We conducted a four-class classification in attempt to separate users into respective GAD severities.

### 3.5. Classification

To validate the selected features, SVM and DT classifiers were used with 10-fold cross-validation to check the veracity of the features using various separations between the labels. SVM and DT classifiers are supervised machine learning algorithms. Our work utilized a one-vs-all approach in conjunction with a linear SVM and a binary classification for DT. Other kernels of SVM such as radial basis function (RBF) and polynomial were tested and we discovered that the linear kernel was able to achieve a similar performance. We were motivated to record the results of the linear kernel due to its explainability and power consumption compared with the alternative kernels. In addition, this manuscript used DT and linear SVM to be consistent with the models used in Nguyen et al. (29).

SVM is a supervised learning method for classification, which is developed through the construction of a set of hyper-planes that separate the respective classes (44). DT is a non-parametric supervised learning method for classification, which predicts the class label through learning simple decision rules from the features. DT can also be represented as a piecewise constant approximation (45).

These classifiers were chosen because of their ability for high performance, high explainability, low complexity, and the given dataset size. SVM and DT offer performance metrics, namely, accuracy, precision, recall, and F1-score. The performance results of the is a fundamental factor for choosing a model. In addition, the chosen models offer high explainability and offer a low complexity.

### 3.6. Performance metrics

Accuracy, precision, recall, and F1 score were used as performance metrics for classification on the selected features (40), as provided in Equations 6, 7, 8 and 9, respectively.

(6) $$\text{Accuracy}=\frac{\text{TN + TP}}{\text{TN + FP + FN + TP}}\;$$

(7) $$\text{Precision}=\frac{\text{TP}}{\text{TP + FP}}\;$$

(8) $$\text{Recall}=\frac{\text{TP}}{\text{TP + FN}}\;$$

(9) $$\text{F1}=2\cdot \frac{\text{Precision}\cdot \text{Recall}}{\text{Precision + Recall}}\;$$

## 4. Results

### 4.1. First phase of COVID-19

After pre-processing, 4,512 samples and 49 features were used for analysis. The samples were separated into GAD severity groups, which include *Minimal* (n=2,609), *Mild* (n=1,218), *Moderate* (n=409), and *Severe* (n=276). Following pre-processing, the proposed feature selection techniques were applied. mRMR was found to achieve the best performance. Our work found that 20 was the optimal number of features required without having to sacrifice the classification accuracy of anxiety severity. The reduced features are described in Table 1.

**Table 1:** Reduced feature set for the First Phase of COVID-19 through mRMR.

Feature	Description	Feature	Description
MH_05	Perceived mental health	LM_40	COVID impact ability meet financial obligations
BH_40D	Eating junk food or sweets	BH_20C	Made plan caring household member are ill
PFSCDV	Household food insecurity	BH_40F	Spending time on the Internet
AGEGRP	Age group	Sex	Sex
MHDVMHI	Perceived mental health derived variable	BH_20M	Other precautions taken to reduce risk
BH_20A	Stocking up on essentials	BH_35C	Exercising outdoors
LM35BCDE	EI benefits (sickness/ caregiver/ worksharing/ other)	BH_40A	Consuming alcohol
RURURB	Rural or urban indicators	BH_110	Number of people in close contact
BH_40E	Watching TV	BH_20D	Making a plan for non-household members
BH_35E	Changing food choices	BH_40B	Using tobacco products

During label separation, the first case separated the classes into *Minimal* and *Severe*, and were classified using a 10-fold SVM and DT, achieving an accuracy of 94.77±0.13% and 92.03±0.24%, respectively. The 10-fold SVM approach achieved a recall, precision, and F1 score of 98.62%, 95.72%, and 97.15%, respectively. To justify the robustness of our approach, this paper used a hierarchical classification approach where the labels were separated between adjacent labels (Figure 3) and tested using an SVM and DT classifier, as shown in Table 2. In the third case, a binary classification with a GAD score cut-off of 10 was conducted. SVM and DT achieved a binary classification accuracy of 86.78±0.15% and 82.82±0.25%, respectively. Lastly, a four-class classification of *minimal, mild, moderate*, and *severe* GAD severities was conducted. The 10-fold SVM and DT achieved an accuracy of 64.65±0.16% and 55.86±0.65%, respectively.

**Table 2:** Hierarchical classification according to class.

Classes	SVM (%)	DT (%)
Minimal vs. mild, moderate, and severe	76.99	68.79
Mild vs. moderate and severe	71.05	62.64
Moderate vs. severe	63.94	57.52

It is also worthwhile to mention that alternative kernels including RBF and polynomial, were also tested for the four cases. The respective results were achieved and can be seen in Table 3. The results of the alternative kernels achieved similar values to the linear SVM kernel. The greater simplicity of the linear SVM further supported our choice of kernel compared with its alternatives.

**Table 3:** Alternative kernels classification per test case for the first phase of COVID-19.

Label separation case	RBF (%)	Polynomial (%)
Minimal vs. severe	93.20	95.80
Hierarchical classification		
Minimal vs. mild, moderate, and severe	74.65	77.91
Mild vs. moderate and severe	67.89	70.60
Moderate vs. severe	55.62	66.13
Binary classification (GAD score of 10)	85.44	90.07
Four-class classification	62.79	58.69

### 4.2. Second phase of COVID-19

After pre-processing, 4,087 samples and 89 features were used for the analysis. The samples were separated into GAD severity groups that included *minimal* (n=2,781), *mild* (n=872), *moderate* (n=285), and *severe* (n=149). Followed by pre-processing, the proposed feature selection techniques were applied. Similar to the first phase, mRMR achieved the best performance. Our work found that 20 was the optimal number of features required without having to sacrifice the classification accuracy of anxiety severity. The reduced feature set is described in Table 4.

**Table 4:** Reduced feature set for the first phase of COVID-19 through mRMR.

Feature	Description	Feature	Description
BH_20D	Making a plan other non-household members	BH_55D	Concerns about the health of Canadian population
BH_40D	Eating junk food or sweets	BH_55A	COVID-19 impact concern on personal health
BH_60C	Frequency of using food delivery service for prepared food (Previous week)	BH_55K	Family stress from confinement
AGEGRP	Age group	Sex	Sex
MHDVMHI	Perceived mental health derived variable	BH_20M	Precautions taken to reduce risk—other
BH_25	General health	FC_20CE	Sources for COVID-19 information accuracy not validated because did not know how to check/too difficult to access
BH_35B	Meditation	BH_20N	Precautions taken to reduce COVID-19 risk—none of the above
RURURB	Rural or urban indicators	IMMIG	Immigration status
BH_40F	Spending time on the Internet	MH_30	General mental health
PBH_110	Number of people in close contact (Yesterday)	BH_20K	Precautions taken to reduce risk by cancelling travels

During label separation, the first case separated the classes into *Minimal* and *Severe* and were classified using a 10-fold SVM and DT, achieving an accuracy of 97.35±0.11% and 96.41±0.20%, respectively. The 10-fold SVM approach achieved a recall, precision and F1 score of 99.03%, 98.39%, and 98.71%, respectively. To justify the robustness of our approach, this paper used a hierarchical classification approach where the labels were separated between adjacent labels (Figure 3) and tested using an SVM and DT classifier, as shown in Table 5. In the third case, a binary classification with a GAD score cut-off of 10. SVM and DT achieved a binary classification accuracy of 91.34±0.06% and 87.27±0.52%, respectively. Lastly, a four-class classification of *minimal, mild, moderate* and *severe* GAD severities was done. The 10-fold SVM and DT achieved an accuracy of 73.38±0.12% and 64.67±0.42%, respectively.

**Table 5:** Hierarchical classification according to class.

Classes	SVM (%)	DT (%)
Minimal vs. mild, moderate, and severe	80.60	74.28
Mild vs. moderate and severe	75.34	65.77
Moderate vs. severe	71.66	63.82

Similar to the first-phase COVID-19 analysis, alternative kernels were tested for the four cases and the respective results can be seen in Table 6.

**Table 6:** Alternative kernels classification per test case for the second phase of COVID-19.

Label separation case	RBF (%)	Polynomial (%)
Minimal vs. severe	91.79	94.71
Hierarchical classification		
Minimal vs. mild, moderate, and severe	71.76	75.04
Mild vs. moderate and severe	65.63	67.08
Moderate vs. severe	58.25	60.83
Binary classification (GAD score of 10)	83.91	86.83
Four-class classification	71.08	66.11

### 4.3. Probability distribution analysis

**Figure 4:**
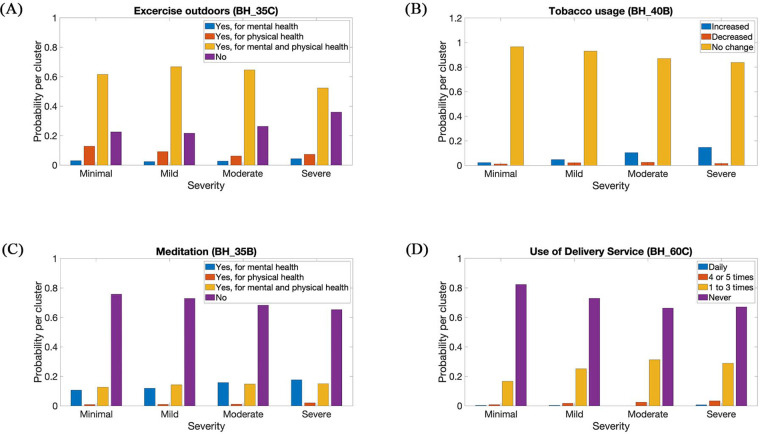
Probability distribution of (**A**) exercises outdoors, (**B**) tobacco usage, (**C**) meditation, and (**D**) use of delivery services, in respect to severity.

The probability distribution for each of the selected features was analyzed, providing support for the selection of the reduced feature set. Figure 4 represents BH_35C, BH_40B, BH_35B, and BH_60C (Table 1). These probability distributions were assessed for each severity level. The resultant probabilities were equal to the number of sample points per response, divided by the total number of samples per severity level. Figure 4A shows a decline in the amount of physical exercise as the severity of anxiety increases. The probability of engaging in physical exercise reduced as the severity of anxiety increased, which matches the finding in Anderson and Shivakumar (46). Figure 4B shows a direct correlation between the severity of anxiety, and the usage of tobacco. Increased levels of anxiety present an increased probability of tobacco usage. This result supports the findings in King et al. (47). Figure 4C shows an increase in the meditation for mental and physical health as anxiety severity increases. There was a mixed response to the effectiveness of meditation in helping reduce anxiety in users (48–51). A potential reason for the increased number of users engaging in meditation might be their attempts to reduce their anxiety level or that they were unsuccessful in their previous meditation attempts due to its various challenges (52). Figure 4D represents the use of delivery services (Daily, 4 or 5 times, 1 to 3 times, and never) in the previous week. The figure outlines an increase in the use of delivery services with an increase in GAD severity. During the COVID-19 pandemic, people may increase their use of delivery services to minimize the risk of being infected (53).

## 5. Conclusion and discussion

The purpose of this work is to analyze the correlates of anxiety symptoms among the Canadian labour force during the first and second phases of COVID-19. This work proposes the use of GAD-7 as the anxiety severity labels, whereas others similar studies used *perceived mental health* (10, 11, 15, 54, 55). The novelty of this work is that we conduct a longitudinal analysis of the first and second phases of COVID-19, whereas Bulloch et al. (27) evaluated GAD severities of only the first phase of COVID-19. The reason for using GAD-7 is that GAD-7 is a psychometrically validated scale for anxiety (24). To the author’s knowledge, this is the first paper to conduct a longitudinal analysis of the first and second phases of COVID-19 CPSS datasets using the GAD-7 survey.

### 5.1. Feature analysis

Pre-processing and feature selection techniques were utilized to reduce the features used from a maximum of 102 to 20 features, in order to improve the efficiency and accuracy of the classifiers. The mRMR algorithm was used to reduce the feature set. Following the analysis of the reduced feature set, it was determined that many of the available features can be augmented as PP data. PP data are qualitative data that can be collected as passive data. For example, within the reduced feature set of the first and second phases of COVID-19 datasets, BH_35B, BH_35C, BH_40A, BH_40B, BH_40C, BH_40D, BH_40E, BH_40F, BH_110/PBH_110, and RURURB can be coined as PP data (Tables 1 and 4). The RURURB dataset is used to determine a participant’s location using the GPS signal, the BH_35C dataset uses an accelerometer for activity recognition, and the BH_40E dataset uses the audio environment to determine if the participant is watching TV. The term PP can be collected through various means, such as digital health devices and wireless and mobile systems. These platforms have the ability to capture PP data in addition to continuous passive data. The passive data can determine user exercise outdoors (BH_35C) as well as offer additional insights such as the frequency, duration, and location of exercises outdoor. Future work can envelope PP to reduce survey fatigue and capture objective measurements.

### 5.2. Classification

During classification, we tested for four cases, namely, *Minimal-Severe*, hierarchical, binary classification (GAD-7 score of 10), and four-class classification. In the first case, the classes *Minimal* and *Severe* were separated. The model used the reduced 20 feature subset and 10-fold SVM for the first phase of COVID-19 and the second phase of COVID-19 to achieve an overall accuracy of 94.77±0.13% and 97.35±0.11%, respectively. We expect to achieve the highest accuracy, when classifying *Minimal* and *Severe*, as the labels are opposite extremes in the GAD-7 severity scale. Given that the classes are represented as the opposite extremes of GAD-7, this a reasonable response that is further supported by our hierarchical classification results.

Our second case employed the hierarchical classification according to Figure 3 as it allows for a granular perspective and comparison between GAD severities. The third case involved a binary test with a GAD score cut-off of 10. This test classifies users into two classes (*Minimal* and *Mild* vs. *Moderate* and *Severe*). The binary test achieved an accuracy of 87.15% and 91.41% for the first and second phases of COVID-19, respectively. Given the high accuracy, the model can give proxy on identifying user anxiety. This gives the potential to augment PP data as it may have the potential to give proxy to user anxiety. This is significant as PP and passive data are more obtainable than active data, as it does not require user input.

Lastly, we classified four classes using 10-fold SVM and DT to achieve an accuracy of 64.65±0.16% and 55.86±0.65% for the first phase of COVID-19, respectively, and 73.38±0.12% and 64.67±0.42% for the second phase of COVID-19, respectively. When comparing the label separations, the four class classifier achieved the lowest accuracies. This was expected as we were classifying more classes and also due to the overlapping features between adjacent classes. GAD is not a black and white separation, as there are common symptoms that users will experience when feeling anxious (56). This is reflected in feelings, behaviours, thoughts, and physical sensation. We can consider anxiety as a spectrum of severities, and therefore, the features of one class, may be common to those of the adjacent classes.

### 5.3. Longitudinal analysis

A comparison of the first and second phases of COVID-19 reveals that we were able to achieve a higher accuracy for *Minimal* and *Severe* separation, hierarchical, and GAD significance for the second phase of COVID-19. Perhaps the reason for this was that the second phase of COVID-19 contained more features, allowing for more perspectives to classify anxiety. In contrast, the first-phase of COVID-19 we were able to achieve a higher four class classification. The reason is that the data were collected during the early stages of pandemic, when users are more mentally healthy. We expect the user population to have lower rates of mental illnesses at the beginning of the pandemic, whereas mental health of users in the general population (57, 58), older individuals (age≥70) (59), and adolescents (mean age=14.4) (60) declined with the onset and progression of the pandemic. Overall, we were able to classify and compare CPSS2 and CPSS4 with relatively high accuracy. Future studies can collect the reduced feature set as EMA for continuous and long-term sampling. The use of EMA will allow increased sampling that can offer more interoperability and to predict the trends of a user’s mental health.

### 5.4. Ethical concerns

The data was collected in accordance with the ethical and privacy principles laid down in the Statistics Act, Revised Statutes of Canada, 1985, Chapter S-19 (61, 62). The datasets used in this study are publicily available and anonymized prior to publication. Anonymization is the process of removing personally identifiable information from data for the purposes of participant confidentiality and privacy. Data has also been volunteered with informed consent and the approval of participants.

### 5.5. Limitations

Because the data are anonymized and confidential, the findings of this paper cannot be applied to a specialized demographic of users. As this paper focuses on the general analysis of anxiety of Canadians, the results between subpopulations may vary.

The model developed used the CPSS data that were collected online through surveys during the pandemic. A limitation of this work is that we had only two datasets that involved the collection of self-perceived anxiety. The longitudinal analysis was conducted on two timestamps. Additional datasets collected at regular intervals or additional time sample points would further enhance the findings and offer a better understanding.

In addition, during the four-label separation, cases can be considered in retrospective analysis. Therefore, the proposed proxy needs to be validated in other datasets and implemented for future studies to determine the capabilities of identifying prospective anxiety in users.

### 5.6. Application

The findings of this work present anxiety severity as increasing from the first phase to the second phase of COVID-19. This implies a general decrease in mental health during the pandemic, which has been confirmed by prior work (18, 27). As previously mentioned, data collection is not continuous, thus making mental health monitoring difficult. However, these models can be applied to similar paradigms using wearables to collect passive data unobtrusively. The use of wearables will allow continuous data collection of similar information that was collected during the CPSS, which can be used to monitor and determine trends of participant mental health over time (63, 64). Future studies can incorporate PP for flexible collection of active data. This would result in lowering survey fatigue and capture of objective measurements. Moreover, this will allow interventions to be developed and orientated around the features studied in this paper. For example, users who increase the tobacco usage due to anxiety episodes can be detected and intervened by systems like mPuff and mobile devices (65). Furthermore, studies can be specialized for subpopulations, allowing better insights and understanding the specific demographics.

With the ability to have an increased sampling of data, we can offer personalized interventions such as ecological momentary interventions, which can be provided to patients in their natural environments (17).

### 5.7. Future work

The commonality between the datasets was limited due to the objectives of Canada Statistics data collection. The common features are related to demographics (RURURB, SEX, AGEGRP) and mental health questions (PBH_110/BH_110, MH_20D, BH_20M, BH_40D, BH_40F, MHDVMHI). Due to the common features, future works can evaluate the effect of demographics on GAD severity for the first and second phases.

The original CPSS surveys contained up to 102 survey questions that can lead to survey fatigue. Survey fatigue is defined as a participant becoming apathetic or bored due to excessive numbers of questions, resulting in the abandonment of the survey. This work reduced the feature set to 20, while also reducing the potential of survey fatigue. The ability to augment the PP data with a passive sensor in combination with efficient classifiers could allow more detailed digital phenotyping. The classification of *Minimal* and *Severe* provides proxy correlates for population anxiety, as well as the ability to prepare and provide interventions accordingly. Moreover, future studies can replicate this work and implement the use of passive and PP features for further analysis of public health policies if they are leading to decreased stress and anxiety in the population. With the presence of COVID-19, mental health has been a common discussion topic. A study of continuous long-term data collection can further explore and understand how people cope during this pandemic.

## Data availability statement

The datasets analyzed for this study can be found in the Canadian Perspectives Survey Series 2: Monitoring the effects of COVID-19, May 2020 and Canadian Perspectives Survey Series 4: Information sources consulted during the pandemic, July 2020.

## Author contributions

BN is the lead author who explored the literature, summarized the findings, developed the machine learning models, summarized the results, and wrote the manuscript. MI helped revise the manuscript. VB is the co-supervisor and offered expertise in psychiatry and assisted with manuscript writing. SK is the principal investigator of this research project, who provided biweekly feedback on the project, and assisted with manuscript writing. All authors contributed to the article and approved the submitted version.

## Funding

This research is funded through Natural Sciences and Engineering Research Council of Canada (NSERC) RGPIN-2020-04628 and Ontario Graduate Scholarship (OGS).
